# The Influence of the Polymer Amount on the Biological Properties of PCL/ZrO_2_ Hybrid Materials Synthesized via Sol-Gel Technique

**DOI:** 10.3390/ma10101186

**Published:** 2017-10-17

**Authors:** Michelina Catauro, Elisabetta Tranquillo, Michela Illiano, Luigi Sapio, Annamaria Spina, Silvio Naviglio

**Affiliations:** 1 Department of Industrial and Information Engineering, University of Campania “Luigi Vanvitelli”, Via Roma 29, 81031 Aversa, Italy; elisabetta.tranquillo@unicampania.it; 2 Department of Biochemistry, Biophysics and General Pathology, Medical School, University of Campania “Luigi Vanvitelli”, Via L. De Crecchio 7, 80138 Naples, Italy; Michela.Illiano@unicampania.it (M.I.); luigi.sapio@unicampania.it (L.S.); annamaria.spina@unicampania.it (A.S.); silvio.naviglio@unicampania.it (S.N.)

**Keywords:** sol-gel technique, biomaterials, cell proliferation, cell cycle

## Abstract

Organic/inorganic hybrid materials are attracting considerable attention in the biomedical area. The sol-gel process provides a convenient way to produce many bioactive organic–inorganic hybrids. Among those, poly(e-caprolactone)/zirconia (PCL/ZrO_2_) hybrids have proved to be bioactive with no toxic materials. The aim of this study was to investigate the effects of these materials on the cellular response as a function of the PCL content, in order to evaluate their potential use in the biomedical field. For this purpose, PCL/ZrO_2_ hybrids containing 6, 12, 24, and 50 wt % of PCL were synthesized by the sol-gel method. The effects of their presence on the NIH-3T3 fibroblast cell line carrying out direct cell number counting, MTT, cell damage assays, flow cytometry-based analysis of cell-cycle progression, and immunoblotting experiments. The results confirm and extend the findings that PCL/ZrO_2_ hybrids are free from toxicity. The hybrids containing 12 and 24 wt % PCL, (more than 6 and 50 wt % ones) enhance cell proliferation when compared to pure ZrO_2_ by affecting cell cycle progression. The finding that the content of PCL in PCL/ZrO_2_ hybrids differently supports cell proliferation suggests that PCL/ZrO_2_ hybrids could be useful tools with different potential clinical applications.

## 1. Introduction

Organic/inorganic hybrid materials are attracting considerable attention today. They are biphasic systems in which the organic and inorganic components are connected on a nanometer scale. A classification of the hybrid materials based on interactions between the phases was proposed by Judenstein and Sanchez [1]. They defined these materials as first and second class hybrids. In particular, Class I consists of organic and inorganic compounds bonded through hydrogen, van der Waals, or ionic bonds, whereas in Class II, the organic and inorganic phases are linked through strong chemical bonds (covalent or polar covalent bonds). Many organic/inorganic hybrid materials can be developed using the sol-gel method. The sol-gel chemistry is based on the hydrolysis and polycondensation reactions of the precursor metal alkoxides M(OR)x, where M represents a network-forming element (such as Si, Sn, Zr, Ti, Al), and R is usually an alkyl group. The sol-gel reactions are dependent on many parameters, such as structure and concentration of the reactants, solvents, and catalysts, as well as reaction temperature and rate of removal of by-products and solvents [2,3]. The sol-gel process provides a convenient way to produce porous [4], biocompatible, and bioactive materials [5]. The last property is due to the presence of –OH groups on the material surface, which can promote apatite layer formation on the biomaterial surface [6]. Therefore, sol-gel materials may be easily osseointegrated when implanted in vivo [7,8]. This is a property required for a material to be used in the dental and orthopedic fields. Moreover, the sol-gel method shows several advantages over most traditional syntheses. It gives homogeneous materials and leads to a fine control of the chemical composition. The low processing temperature allows one to add thermolabile molecules, such as polymers, drugs, and/or biomolecules, to the glassy matrix [9], obtaining organic/inorganic hybrids. The main idea, when a hybrid material is developed, is to overcome the disadvantages of each of the components and to retain their advantages. Among the synthetic polymers, poly(e-caprolactone) (PCL), which is a biodegradable aliphatic polyester [10,11,12], has already been proposed for many biomedical applications, such as drug delivery and tissue engineering [12,13,14,15,16,17,18,19,20,21]. However, PCL is too flexible and weak to satisfy the mechanical requirements for this specific application, while polymer-based nanocomposite materials provide an alternative choice to overcome these problems [22,23,24,25]. On the other hand, it is known that glass matrices, such as zirconia, are bioactive and biocompatible [26,27], but they cannot be used in some applications due to their poor mechanical properties.

Elsewhere, Catauro et al. proposed the use of organic/inorganic hybrid materials based on zirconia (ZrO_2_), with different percentages of PCL embedded, for biomedical applications [28]. These materials were extensively characterized [29]. The presence of hydrogen bonds between the carboxylic groups of the polymer and the hydroxyl groups of the inorganic matrix was proved by Fourier transform infrared (FTIR) analysis, and is strongly supported by solid-state NMR [29]. SEM analysis confirmed that they can be considered hybrid materials and no appreciable difference between their morphologies was observed [29]. Furthermore, the materials are nanocomposites, as shown by AFM analysis [30]. XRD diffractograms showed that ZrO_2_/PCL gel exhibits broad humps that are characteristic of amorphous materials, even though the PCL diffractogram showed the sharp peaks typical of a crystalline material [31].

PCL/ZrO_2_ hybrids were proposed as matrices for the controlled release of drugs [30], or as coatings able to improve the biological properties of bio-inert titanium implants or to release drugs in a controlled manner [28,32]. The obtained films, indeed, appeared to be more bioactive and biocompatible than uncoated titanium. The coatings induced the formation of hydroxyapatite when soaked in SBF, did not show cytotoxicity and were also supportive of cell proliferation at all compositions. In order to evaluate the potential use of such materials as biomaterials, further knowledge about their biological properties is needed. Therefore, the aim of the present study has been to investigate, more in depth, the effects of the presence of PCL/ZrO_2_ materials as a function of the PCL content on the NIH-3T3 fibroblast cellular response and the underlying mechanisms. Therefore, five PCL/ZrO_2_ hybrid samples containing different amount of PCL (6, 12, 24, and 50 wt %) have been synthesized via the sol-gel route as reported elsewhere [29] and the effects of their presence on cell viability and cell cycle progression, as well as membrane integrity have been evaluated. All of the results are reported as a function of PCL content.

## 2. Results and Discussion

### 2.1. PCL/ZrO_2_ Hybrid Materials Do Not Have Cytotoxic Effects

The mouse NIH-3T3 cell line is a well-established model system of fibroblasts, largely used to study cellular responses, including those induced by biomaterials [33,34,35,36,37,38].

**Figure 1:**
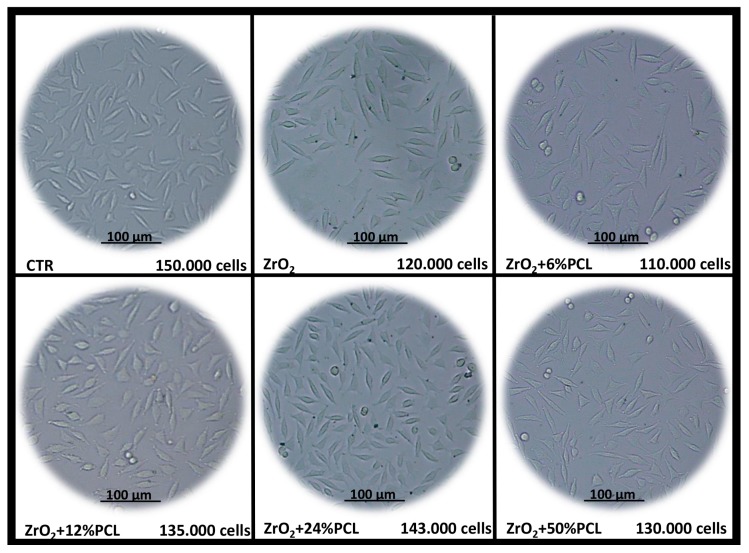
Typical phase-contrast images of NIH 3T3 cells treated with and without PCL-free ZrO_2_ material and poly(e-caprolactone) (PCL)/ZrO_2_ hybrids with different percentages of PCL (6 wt %, 12 wt %, 24 wt %, 50 wt %) for 48 h.

In order to evaluate the cytotoxicity of the bioactive PCL/ZrO_2_ hybrid materials (synthesized as shown in the Materials and Methods section) as a function of PCL content, NIH-3T3 fibroblasts were grown in the absence and presence of the pure ZrO_2_ and PCL/ZrO_2_ hybrid materials, each containing different percentages (6, 12, 24, 50 wt %) of PCL, for up to 72 h. Over the time course, the plates were observed daily under phase-contrast microscopy and relative pictures were taken (Figure 1). Notably, in Figure 1 it is shown that the cells grown in the presence of our preparations appeared not to suffer and shared a morphology similar to that of control untreated cells. In addition, it is also observed that the 12% and 24% PCL/ZrO_2_ hybrids treated plates contain more cells as compared to plates treated with PCL-free ZrO_2_, 6% and 50% PCL/ZrO_2_ hybrids, with a cell density similar to that of control untreated plate.

**Figure 2:**
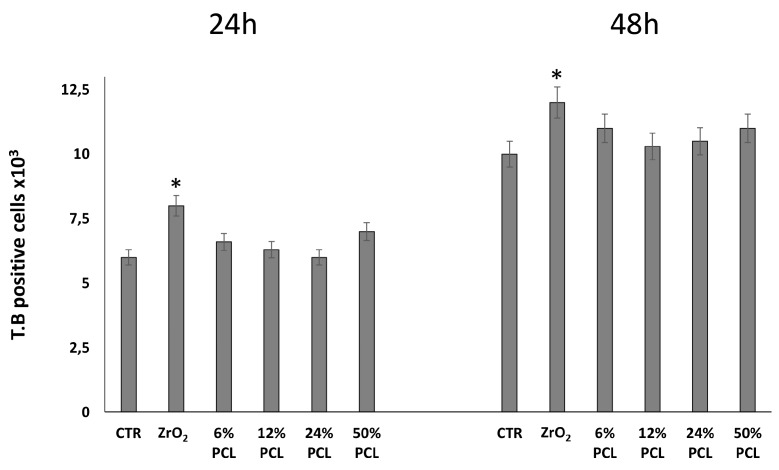
Trypan Blue Exclusion Dye test. NIH-3T3 cells were cultured in 10% serum containing medium in the absence (control, CTR) or presence of PCL-free ZrO_2_ and PCL/ZrO_2_ hybrid materials with different percentages (6 wt %, 12 wt %, 24 wt %, 50 wt %) for 24 and 48 h. Then, the cell number was recorded as stated in Materials and Methods. Data represent the average of three independent experiments. The means and S.D. are shown. *, *p* < 0.05 vs. control cells.

Then, cells were detached from monolayers by brief exposure to a solution of trypsin and a Trypan Blue dye exclusion assay was performed (Figure 2). Interestingly, Figure 2 shows that the number of Trypan Blue positive dead cells increased in the presence of pure ZrO_2_ (≈15%) as compared to control untreated cells, with less evident variations in the presence of PCL/ZrO_2_ hybrid materials.

Finally, we recovered the culturing medium in which NIH-3T3 fibroblasts were grown, and tested it, by using the Abbott Lab Chemistry Analyzer ci 8200 (usually used to test blood samples), looking for the presence of any known intracellular enzymatic activities, such as lactate dehydrogenase, (LDH), aspartate aminotransferase (AST), and alanine aminotransferase (ALT) [39,40], to evaluate their release upon possible cell damage in response to the materials (Table 1). The results, reported in Table 1, show a clear increase of both LDH and AST activities in media from NIH-3T3 fibroblasts treated with the cytotoxic agent doxorubicin (DOXO) (1 µM) (used as positive control) [41] as compared to the untreated ones. Moreover, media from plates without cells were tested as a negative control. In addition, total proteins and ALT transaminase were measured as further internal controls [40]. As expected, according to the absence of ALT activity in fibroblasts [42,43], no increase of such activity is seen in any samples.

Remarkably, Table 1 shows that no increase of AST transaminase activity is detected in medium from NIH-3T3 fibroblasts grown for 72 h in the presence of PCL/ZrO_2_ hybrid materials, as compared to the PCL-free ZrO_2_, whereas only a very slight increase of LDH activity (from 61 to 64 U/L) is recorded. This results suggests that minimal, if any, cell membrane injury occurs in response to PCL/ZrO_2_ hybrid materials.

Overall, the above data clearly indicate that the content of PCL in the PCL/ZrO_2_ hybrid materials does not cause cell damage on NIH-3T3 fibroblasts.

### 2.2. PCL Content in the PCL/ZrO_2_ Hybrids Positively Affects the Cell Proliferation

**Figure 3:**
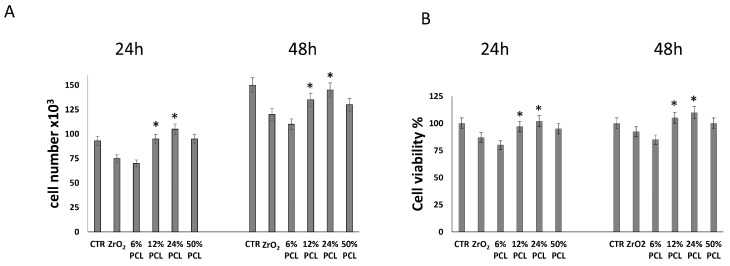
Effects of PCL-free ZrO_2_ material, PCL/ZrO_2_ hybrid materials with different percentages (6 wt %, 12 wt %, 24 wt %, 50 wt %) of PCL on cell proliferation. NIH-3T3 cells were cultured in 10% serum containing medium in the absence (control, CTR) or presence of the indicated substances for 24 and 48 h. Then, the cell number was recorded (**A**); and cell viability was measured by MTT assay (**B**). Data represent the average of three independent experiments. The means and S.D. are shown. * *p* < 0.05 vs. PCL-free ZrO_2_ treated-cells.

To test the influence of PCL content on cell proliferation, NIH-3T3 fibroblasts were grown for 24 and 48 h in 10% serum containing medium in the absence (control cells) or presence of the following preparations: PCL-free ZrO_2_ and PCL/ZrO_2_ hybrid materials with different percentages (6 wt %, 12 wt %, 24 wt %, 50 wt %) of PCL. Thereafter, cell proliferation was determined by direct cell number counting and by conventional MTT assay (Figure 3).

Figure 3 shows that the cell population is significantly higher, at both time points, in plates containing 12 wt % and 24 wt % PCL/ZrO_2_ hybrid materials, as compared to cell plates cultured in the presence of pure ZrO_2_, whereas no significant variations in cell number are evident in the presence of 6 wt % and 50 wt % PCL/ZrO_2_ hybrid materials. In agreement with previous findings [34,44], a decrease of cell number and cell viability in response to PCL-free ZrO_2_ is evident as compared to control fibroblasts (grown in the absence of materials), according to a growth impairment induced by such material. Recently, indeed, Meesser et al. [44] proved that most ceramics cause a mild suppression of cell functions in vitro. However, the levels of the suppression observed would be acceptable on the basis of standards used to evaluate alloys and composites (<25% suppression of dehydrogenases activity).

Altogether, the above results suggest that the PCL/ZrO_2_ hybrids, mainly those containing 12 wt % and 24 wt % PCL, positively affect cell proliferation when compared to the inorganic material in the non-hybrid form. Our data are in agreement with previous similar findings based mainly on cell viability MTT assay [28,34], supporting the evidence that the presence of different PCL amounts favors the preparation of biomaterials with improved biocompatibility. In fact, it is known that its slow degradation kinetics, biocompatibility, and semicrystalline nanofibers can result in stimulation of the extracellular matrix providing a good scaffold for cell proliferation and engineering [25,45].

**Figure 4:**
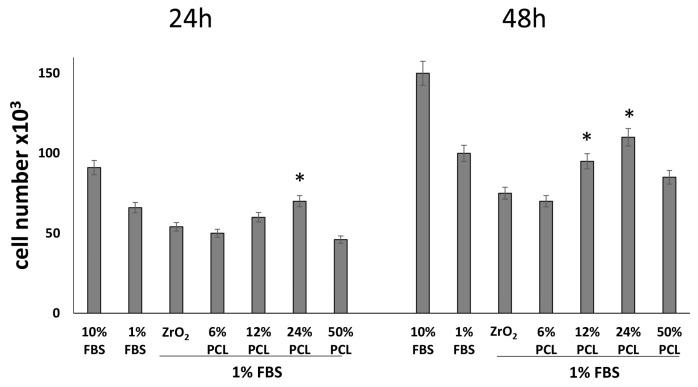
Effects of pure ZrO_2_, PCL/ZrO_2_ hybrid materials with different PCL percentages (6 wt %, 12 wt %, 24 wt %, 50 wt %) in low serum (1%) containing medium on cell proliferation. NIH-3T3 cells were cultured in low serum (1%) containing medium in the absence (control) or presence of the indicated substances for 24 and 48 h. Then, the cell number was recorded. Data represent the average of three independent experiments. The means and S.D. are shown. * *p* < 0.05 vs. PCL-free ZrO_2_ treated-cells.

The slightly lower positive effect observed in the plate containing the system with 6 wt % PCL as compared to those ones containing 12 wt % and 24 wt % suggests that when the polymer is added at a low level, the effect of the ZrO_2_ matrix predominates. Probably, the inorganic network masks the polymer that is embedded and linked within it by the formation of –OH bonds. On the other hand, the decrease of the positive effect recorded when the PCL content is higher than 24%, is in agreement with other works in literature [46,47], which ascribed the effect of slight inhibition to the hydrophobic nature of PCL inhibitings cells adhesion. This, in turn, hinders cell proliferation, and thus causes a decrease of cells vitality. However, it has been proven [48] that the addition of hydrophilic SiO_2_ to PCL coatings leads to an increase of coating hydrophilicity. Therefore, up to 24 wt % of PCL the presence of zirconia matrix make the synthesized hybrid materials hydrophilic and the negative effect of PCL is observed only for higher polymer amounts. The dependence between cell proliferation and the PCL content can be due to a different degradation rate of the polymer in the different hybrid materials. It is reported in literature that a high PCL degradation rate negatively affects cell proliferation [22,23,24,25] and when 50 wt % of polymer is added in the zirconia matrix, part of the PCL cannot forms H-bonds because all hydroxyl groups of the matrix are already involved in H-bonds. Therefore, the polymer can be highly subjected to degradation process. This is in accordance with the results of Vecchio Ciprioti et al.[49], who showed the protective role of the zirconia matrix in ZrO2/PEG hybrid materials against the thermal degradation of the polymer. To increase the specificity and reliability of the above data, we tested the effects of the five formulations on NIH-3T3 cell proliferation, also in a different experimental growth condition, i.e., by culturing fibroblasts in low serum (1%) containing medium for 24 and 48 h. As expected, in all 1% serum samples we found less cells when compared to the 10% serum samples (Figure 4) [50]. Interestingly, Figure 4 also shows that the cell population again is higher in plates containing 12 wt % and 24 wt % PCL/ZrO_2_ hybrid materials, as compared to cell plates cultured in presence of the PCL-free ZrO_2_.

Overall, these data confirm that even in low serum conditions, i.e., in adverse growth conditions, no deleterious cytotoxic effect due to PCL content in PCL/ZrO_2_ hybrid materials could be seen and also that the hybrids with PCL percentages of 12 wt % and 24 wt % result in a growth advantage to NIH-3T3 fibroblasts.

### 2.3. PCL Content in the PCL/ZrO_2_ Hybrids Affects the Cell Cycle Progression

**Figure 5:**
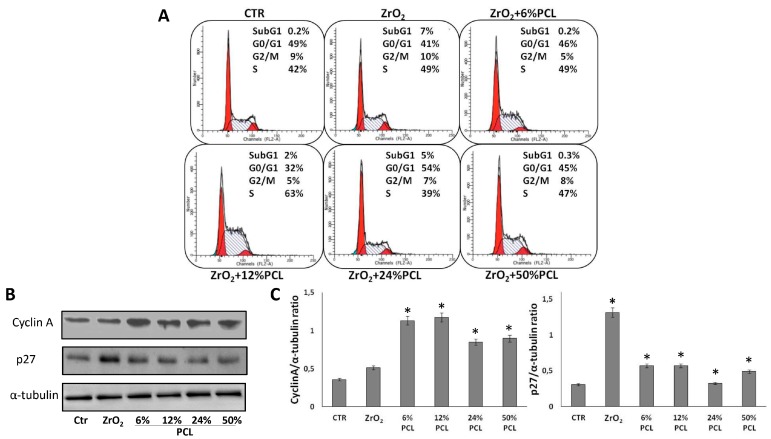
Effects of ZrO_2_ material alone and PCL/ZrO_2_ hybrid materials with different PCL percentages (6 wt %, 12 wt %, 24 wt %, 50 wt %) on the distribution of NIH-3T3 cells in cell cycle phases and on the levels of some relevant cell cycle regulating proteins. NIH-3T3 cells were cultured in the absence (control, CTR) or presence of the indicated substances for 48 h. (**A**) FACS analysis of propidium iodide stained cells. Profiles and quantitative data indicating the percentage of G0/G1, S, and G2/M cells from a typical representative experiment; (**B**) 30 μg of cell extracts were subjected to SDS-PAGE and blotted with antibodies against the indicated proteins (α-tubulin was used as a standard for the equal loading of protein in the lanes). The image is representative of two immunoblotting analyseis from two different cellular preparations with similar results; (**C**) Graphs showing the densitometric intensity of Cyc A and p27/tubulin bands ratio. The intensities of signals were expressed as arbitrary units. * *p* < 0.05 vs. control untreated cells.

To further explore the influence of PCL content on NIH-3T3 cell proliferation, the distribution of cells in the G0/G1, S, G2/M cell cycle phases was evaluated by flow cytometric analysis of propidium iodide-stained cells. (Figure 5). The sub-G1 population (i.e., the proportion of cells with hypoploid DNA content), characteristic of cells having undergone DNA fragmentation (the biochemical hallmark of apoptotic cell death) was also looked at. Firstly, Figure 5 shows that the percentages of asynchronously growing control NIH-3T3 fibroblasts in the G0/G1, S, and G2/M phases are 49%, 42%, 9%, respectively, in full agreement with previous result [51]. Notably, in agreement with the described weak cytotoxic effect of PCL-free ZrO_2_ [34,44] (Figure 2), in Figure 5 the appearance of a sub-G1 cell death population (7%) was observed in ZrO_2_ pure treated cells, as compared to control untreated cells, whereas such a sub-G1 population is either completely absent or only present to a low extent in PCL/ZrO_2_ hybrid materials, suggesting a protective/growth supporting effect of the PCL component. In addition, interestingly, fibroblasts grown in the presence of 12 wt % PCL/ZrO_2_ hybrid materials accumulate more in the S phase (62%), whereas fibroblasts grown in the presence of 24 wt % PCL/ZrO_2_ hybrid materials have a more evident G0/G1 phase (53%) compared to those cultured in the presence of PCL-free ZrO_2_, suggesting that the PCL content can differently affect cell cycle progression. These results are in accordance with the data obtained via cell counting and the MTT assay. The different modification of the cell cycle progression, indeed, may be attributed to the different concentration of the PCL degradation products in the medium, due to the different PCL degradation rate in the hybrid system [52,53]. This, in turn, can differently affect the pathway(s) involved in the progression of the cell cycle [22,23,24,25].

To obtain further insights into the possible effects of PCL/ZrO_2_ hybrid materials on the cell division cycle of NIH-3T3 cells, we studied the expression of some relevant cell cycle regulating proteins in response to the following preparations: ZrO_2_ and PCL/ZrO_2_ hybrid materials with different percentages (6 wt %, 12 wt %, 24 wt %, 50 wt %) of PCL (Figure 5B).

To this aim, NIH-3T3 cells were exposed to the materials for 48 h. The cell extracts were analyzed by Western blotting to examine the levels of cyclin-dependent kinase inhibitor p27 and of cyclin A proteins.

Remarkably, it was noted that cyclin A and p27 protein levels, as well as those of other relevant cell cycle proteins, oscillate depending on cell cycle phases and are strongly involved in cell cycle progression [50,54,55]. In Figure 5B it is shown that the amounts of the positive cell cycle regulator cyclin A and of cell cycle inhibitor p27 increased and decreased, respectively, in response to PCL/ZrO_2_ hybrid materials. Moreover, there is evidence that p27 (that is a cell cycle inhibitor) is more abundant in ZrO_2_ pure treated cells when compared to control untreated cells and decreases in response to hybrids, whereas cyclin A (that is a positive regulator of cell cycle) increases (Figure 5B). Interestingly, this is in agreement with the growth promoting effect of the hybrid materials, as compared to PCL-free ZrO_2_ which, on the contrary, has a growth inhibitory effect. Overall, the above cell cycle and Western blot data suggest that 12 wt % and 24 wt % PCL/ZrO_2_ hybrid materials affect the cell cycle progression of NIH-3T3 cells more than the others compositions.

## 3. Materials and Methods

### 3.1. Sol-Gel Synthesis

The organic/inorganic PCL/ZrO_2_ hybrids materials, containing 0, 6, 12, 24, and 50 wt % of the organic component, were synthesized by the sol-gel process as elsewhere [29]. A zirconium(IV) propoxide solution (Zr(OC_3_H_7_)_4_ 70 wt % in n-propanol, Sigma Aldrich) and PCL (Mn = 10,000) were used as precursors of the inorganic and organic phases, respectively. A solution of PCL in chloroform was added to the solution of Zr(OC_3_H_7_)_4_ in an ethanol–acetylacetone–water mixture. Acetylacetone was used to control the hydrolytic activity of zirconium alkoxide. The solution was stirred by a magnetic stirrer until the resulting sols were uniform and homogeneous. The molar ratios between the reagent achieved were: CH_3_CH_2_OH/Zr(OCH_2_CH_2_CH_3_)_4_ = 5.7; Zr(OCH_2_CH_2_CH_3_)_4_/AcAc = 4.5.

**Figure 6:**
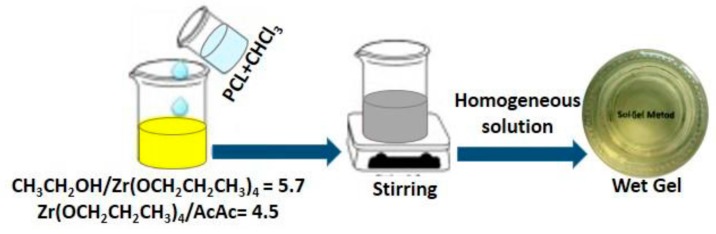
Flow chart of hybrids synthesis by the sol-gel method and the molar ratios between the reagents achieved in the sol.

After gelation, all of the wet gels were air dried at 45 °C for 48 h to remove residual solvents without any polymer degradation. As indicated in the text, Figure 6 shows the flow chart of hybrid synthesis by the sol-gel method.

### 3.2. Cell Culture and Treatments

The mouse fibroblast NIH-3T3 cell line was obtained from the American Type Culture Collection (Rockville, MD, USA). NIH-3T3 cells were grown in Dulbecco′s Modified Eagle′s Medium (DMEM) supplemented with 2 mM glutamine, 100 U/mL penicillin, 100 mg/mL streptomycin, and 10% fetal bovine serum (FBS), and cultured at 37 °C in a 5% CO_2_ humidified atmosphere. Typically, cells were split (5 × 10^5^/10 cm plate, 6 × 10^4^/6-well plate) and grown in 10% serum containing medium. After 24 h, the medium was removed, the cells were washed with PBS and incubated with 10% (in some experiments 1%) FBS fresh medium (Time 0), in the absence (control cells) or in the presence of the organic/inorganic PCL/ZrO_2_ hybrid materials, at 0.3 mg/mL concentration, containing 0, 6, 12, 24, and 50 wt % of the organic component PCL and grown for the times indicated in the text and figures. After treatment, both floating cells and adherent cells were recovered and counted (by centrifugation from culture medium and harvested by trypsinization, respectively). In the case of experiments designed for fluorescence-activated cell sorting (FACS) analysis and the evaluation of the level of proteins involved in cell cycle progression, adherent cells were collected, washed twice with ice-cold PBS, and divided into two aliquots (see below).

### 3.3. Trypan Blue Exclusion Test

The Trypan Blue (dye) exclusion test is used to determine the number of dead cells present in a cell suspension. Briefly, cells were seeded in 10% PBS-containing medium in a 6-well plate at a density of 6 × 10^4^ cell/well for 24 and 48 h at 37 °C and cultured, as above. At the indicated times, after cell collection, cell counting was performed by mixing 10 μL of cell suspension with an equal volume of Trypan Blue (TB; 0.4%, *v/v*). The number of blue staining cells (dead cells) and of viable cells (that exclude the Trypan Blue) was recorded. TB experiments were performed three times (in replicates of six wells for each data point in each experiment). Data are presented as means ± standard deviation for a representative experiment.

### 3.4. Cell Viability Assay

Cell viability assay was performed, as described previously [41]. Briefly, cells were seeded in 10% PBS-containing medium in a 96-well plate at a density of 3 × 10^3^ cells/well for 24 and 48 h at 37 °C and cultured, as above. Viable cells were determined by the 3-[4,5-dimethylthiazol-2-yl]-2,5-diphenyltetrazolium bromide (MTT) assay and cell viability was assessed by adding MTT solution in PBS to a final concentration of 0.5 mg/mL. The plates were then incubated at 37 °C for 4 h and the MTT-formazan crystals were solubilized in a solution of 4% 1N isopropanol/hydrochloric acid at 37 °C on a shaking table for 20 min. The absorbance values of the solution in each well were measured at 570 nm using a Bio-Rad 550 microplate reader (Bio-Rad Laboratories, Milan, Italy). Cell viability was expressed as a percentage of absorbance values in treated samples with respect to that of control (100%). MTT experiments were performed three times (in replicates of 6 wells for each data point in each experiment). Data are presented as means ± standard deviation for a representative experiment.

### 3.5. Evaluation of Cell Cycle Phases by Flow Cytometry

After treatment, the cells were recovered as previously described in “Cell culture and treatments”, fixed by resuspension in 70% ice-cold methanol/PBS and incubated overnight at 4 °C. The cells were then centrifuged at 1200 RPM ((revolutions per minute) for 5 min, and the pellets obtained were washed with ice-cold PBS, centrifuged for 5 min, resuspended in 0.5 mL DNA staining solution (50 μg/mL PI and 100 μg RNase A in PBS), and incubated overnight at 37 °C in the dark. The samples were then transferred to 5-mL Falcon tubes and stored oin ice until assayed. Flow cytometry analysis was performed using a FACSCalibur flow cytometer (Becton Dickinson, San Jose, CA, USA) interfaced with a Hewlett-Packard computer (model 310) for data analysis. For the evaluation of intracellular DNA content, at least 20,000 events for each point were analyzed, and regions were set up to acquire quantitative data of cells that fell into the normal G1, S, and G2/M regions [50]. Results were analyzed using ModiFIT Cell Cycle Analysis software (Version 3.0, verity software house, Inc., Topsham, ME, USA).

### 3.6. Preparation of Cell Lysates

Cell extracts were prepared as previously described [41]. Briefly, after treatments, the cells were recovered as previously described in “Cell culture and treatments”; 3–5 volumes of RIPA buffer (PBS, 1% NP-40, 0.5% sodium deoxycholate, 0.1% SDS) containing 10 μg/mL aprotinin, leupeptin, and 1 mM phenylmethylsulfonyl fluoride (PMSF) were added to the recovered cells. After incubation on ice for 1 h, samples were centrifuged at 18,000 g in an Eppendorf microcentrifuge for 15 min at 4 °C and the supernatant (SDS total extract) was recovered. Some aliquots were taken for protein quantification according to the Bradford method; others were diluted in 4× (to be used at 1:4 dilution) Laemmli buffer, boiled and stored as samples for immunoblotting analysis.

### 3.7. Western Blot Analysis

Typically, we employed 20–40 μg of total extracts for immunoblotting [56]. Proteins from cell preparations were separated by sodium dodecyl sulphate-polyacrylamide gel electrophoresis (SDS-PAGE) and transferred onto nitrocellulose sheets (Schleicher & Schuell, Dassel, Germany) by a Mini Trans-Blot apparatus (BioRad, Hercules, CA, USA). Membranes were washed in TBST (10 mM Tris, pH 8.0, 150 mM NaCl, 0.05% Tween 20), and were blocked with TBST supplemented with 5% nonfat dry milk. Then the membranes were incubated with different primary antibodies in TBST and 5% nonfat dry milk, washed, and incubated with horseradish peroxide-conjugated secondary goat anti-rabbit or anti-mouse antibodies, conjugated with horseradish peroxidase (BioRad). Enhanced chemiluminescence detection reagents were used as a detection system (ECL), according to the manufacturer’s instructions (Amersham Biosciences, Little Chalfont, UK).

### 3.8. Statistical Analysis

Most of the experiments were performed at least three times with replicate samples, except where otherwise indicated. Data are plotted as mean ± SD (standard deviation). The means were compared using analysis of variance (ANOVA) plus Bonferroni’s *t*-test. *p* values of less than 0.05 were considered significant.

## 4. Conclusions

Organic/inorganic hybrid materials represent a very attractive field in biomedical area. The sol-gel method provides a versatile way to produce bioactive hybrid materials and many organic/inorganic hybrids, including poly(e-caprolactone)/Zirconia (PCL/ZrO_2_), have been developed by the sol-gel process.

In this study, we describe by multiple assays (including those aimed to test the release of intracellular enzymatic activities such as transaminases and lactate dehydrogenase upon cell damage), that PCL/ZrO_2_ hybrid materials are free from toxicity. We also provide interesting evidence that the hybrids containing 12 wt % and 24 wt % PCL, more than 6 wt % and 50 wt % ones, enhance cell proliferation when compared to PCL-free ZrO_2_, and impacted cell cycle progression.

The underlying molecular mechanisms by which PCL/ZrO_2_ hybrid materials support cell cycle progression and cell proliferation, remain unclear and need to be investigated. It is to be noted that we are planning this for future studies. Overall, whatever the exact mechanism(s), our results enrich the evidence of favourable biological properties of PCL/ZrO_2_ hybrid materials and suggest the further development of such hybrids for dental and orthopedic applications.

## Figures and Tables

**Table 1:** Effects of pure ZrO_2_ material, PCL/ZrO_2_ hybrid materials with different percentages (6 wt %, 12 wt %, 24 wt %, 50 wt %) of PCL, and doxorubicin on the release of AST, ALT, LDH intracellular enzymatic activities. NIH-3T3 cells were cultured in the absence (control) or presence of the indicated substances for 72 h. Then, the medium from cultured cells plates and “blank” plates was collected and tested for the indicated parameters. Values are means ± S.D. of triplicate samples of a typical experiment. *, *p* < 0.05 hybrids vs. PCL-free ZrO_2_ treated-cells and Doxo vs. control.

Sample Labels	Total Proteins g/dL	AST U/L	ALT U/L	LDH U/L
Blank	1.0 ± 0.05	3 ± 0.1	6 ± 0.2	54 ± 1
Control	1.1 ± 0.04	5 ± 0.1	6 ± 0.1	58 ± 2
ZrO_2_	1.1 ± 0.03	6 ± 0.3	6 ± 0.2	61 ± 3
6 wt % PCL	1.0 ± 0.03	5 ± 0.2	6 ± 0.1	64 ± 2 *
12 wt % PCL	1.1 ± 0.02	5 ± 0.3	6 ± 0.3	63 ± 3
24 wt % PCL	1.1 ± 0.05	6 ± 0.2	6 ± 0.4	62 ± 3
50 wt % PCL	1.0 ± 0.02	5 ± 0.1	6 ± 0.1	64 ± 2 *
DOXO	1.1 ± 0.05	23 ± 1 *	6 ± 0.2	130 ± 5 *
